# Neutrophil Volume, conductivity and scatter (VCS) as a screening tool in neonatal sepsis

**DOI:** 10.1038/s41598-020-61434-z

**Published:** 2020-03-10

**Authors:** Prerana Nesargi, H. S. Niranjan, Prathik Bandiya, Naveen Benakappa

**Affiliations:** 1 Kidwai memorial institute of oncology, Bangalore, India; 2 Indira Gandhi institute of child health, Bangalore, India

**Keywords:** Biochemistry, Neonatology

## Abstract

The initial evaluation of a suspected sepsis in a neonate is always challenging. There are many methods to screen a neonate with suspected sepsis. One of newer method is to assess the changes in neutrophil volume conductivity and scatter. The objective of this study was to establish changes in Neutrophil volume conductivity scatter (VCS) in neonatal sepsis and to determine appropriate cut off levels using receiver operating characteristic (ROC) curves. Neonates with suspected sepsis were evaluated with blood counts, culture and neutrophil VCS parameters. Based on these parameters neonates were classified into sepsis group (Blood culture positive), Probable sepsis group (clinical course consistent with sepsis and positive sepsis screen and negative blood culture), No sepsis group (Clinical course not suggestive of sepsis with negative sepsis screen and blood culture). A total of 304 neonates were included in the study of which 144 were in sepsis group and 160 in no sepsis group respectively. Among the neutrophil VCS parameters there was significant difference between the groups with respect to mean neutrophil volume (MNV) and volume distribution width (VDW) (180 vs 163 vs 150) (p < 0.01). MNV and VDW had good sensitivity (95%, 82%) and specificity (86%, 74%) for diagnosis of sepsis. In conclusion, Neutrophil VCS parameters, especially MNV, can be incorporated with other sepsis screen parameters in diagnosis of neonatal sepsis.

## Introduction

Neonatal sepsis is one of the leading cause of mortality in developing countries^1^. One of the most important challenges faced by neonatologists is accurate early diagnosis of neonatal sepsis. Improper diagnosis has led to unnecessary abuse of antibiotics and alarmingly increase in antibiotic resistance in developing countries^2^.

The conventional tests, commonly called sepsis screen, most often ordered in this scenario are labour intensive time consuming and subjective because of human interpretation^3^.

The Volume, Conductivity and Scatter (VCS) technology of coulter LH780 haematology analyser (Beckman coulter, fullerton, CA) can obtain data from WBCs and these VCS parameters can detect morphological changes in immature and reactive neutrophils. This technology is similar to evaluation of peripheral blood smear by microscope but it evaluates more than a microscope^4^.

It measures cell volume (mean neutrophil volume, MNV), conductivity (mean neutrophil conductivity, MNC) and scatter using direct current impedence, radiofrequency opacity and laser beam respectively^5^.

The information about cell size is given by MNV and internal chemical composition of the cell, especially the nuclear density and cytoplasmic granularity by MNC^5^.

This technology gives results in minutes with no additional blood needed than for the Complete Blood Count^6^.

Neutrophil VCS parameters were evaluated to diagnose sepsis and were found useful in adult population and it seems to be promising and cost effective^4^. Hence this study was planned to evaluate its role as a screening parameter in neonatal sepsis.

## Objectives

The objective of the study was to establish changes in Neutrophil VCS in neonatal sepsis and to determine appropriate cut off levels using receiver operating characteristic (ROC) curves.

## Methods

This was a prospective observational study conducted over a period of one year from January 2015 to December 2015 in Indira Gandhi Institute of Child health, a tertiary care centre in south India.

All the neonates with maternal risk factors for sepsis (in suspected early onset sepsis) or clinical signs and symptoms suggestive of sepsis were included in the study. Neonates who had received antibiotics for more than 2 days, non salvagable at the time of admission as decided by the investigator, perinatal asphyxia, gestational hypertension, convulsion in past 6 hours and blood group incompatibility were excluded from the study. Informed consent was obtained from the parents of all the neonates. After inclusion, blood counts, C- reactive protein (CRP) and blood culture were performed in all clinically suspected cases of sepsis, before starting antibiotics. Among the cases with maternal risk factors for sepsis, blood culture was drawn before starting antibiotics and blood counts and CRP was sent at 6 hours of life. Repeat blood counts and CRP was done after 24 hours in cases of negative sepsis screen. Blood counts were processed in the automated hematology analyser, Beckman coulter LH 750 which gives the neutrophil VCS parameter values. Neutrophil VCS parameters like MNC (mean neutrophil conductivity), CDW (conductivity distribution width), VDW (volume distribution width) MNS (mean neutrophil scatter), SDW (scatter distribution width) and VCS (volume conductivity scatter) were analysed. Blood culture was performed by automated BACTEC method (Bact/ Alert 3D, BioMérieux, Durham, NC, USA) and CRP was performed by immunoturbidometry.

All methods were carried out in accordance with relevant guidelines and regulations. The study was approved by institutional ethical committee of Indira Gandhi Institute of child health, Bangalore.

All the results of the investigations were recorded in the predesigned proforma. Subsequently neonates were classified into three group based on the following group definitions

### Culture positive sepsis group (S)

Neonates with positive blood culture.

### Probable sepsis group (Ps)

Neonates with clinical course consistent with sepsis and with elevated CRP/abnormal blood counts/peripheral blood smear having changes suggestive of infection but with negative culture.

### No sepsis (Ns)

Neonates with clinical course not suggestive of sepsis with sterile blood culture and normal CRP, sepsis screen and peripheral smear.

## Statistical Analysis

All analysis were performed using IBM SPSS Statistics 20.0 Software. Results were expressed as the median and IQR as the data was not normal. Comparisons between median values of the groups were performed by ANOVA (analysis of variance).

Non- Parametric tests ie Kruskal -Wallis test, and multiple comparisons were performed in pairs to determine which mean values differ. A two-tailed-Mann-Whitney U test was performed in which p value <0.05 was considered statistically significant.

The diagnostic properties of each test were investigated by receiver operator characteristic (ROC) curves. The optimum cut-off value for each variable was tested. Subsequently, the sensitivity, specificity and area under the ROC curve were assessed.

## Results

**Figure 1:**
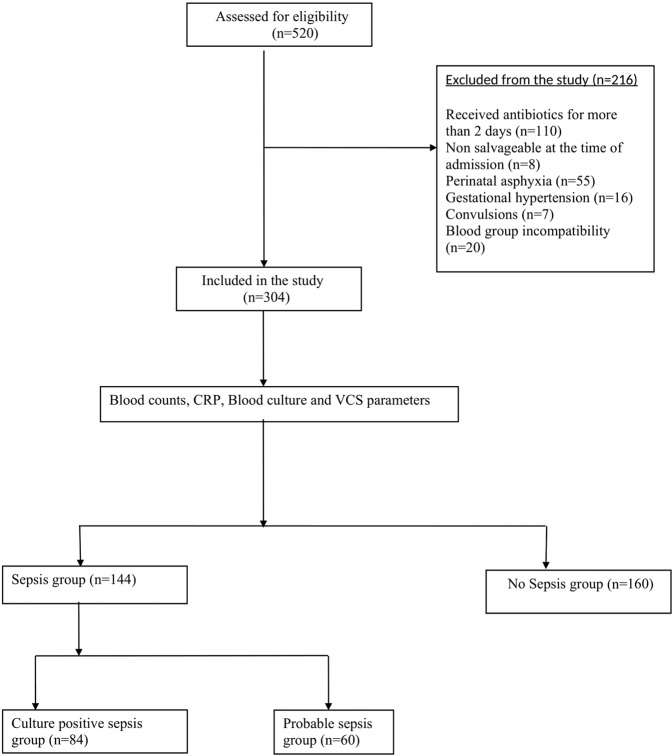
Study flow diagram.

A total of 520 neonates were eligible for the study of which 216 neonates were excluded for various reasons. After exclusion 304 neonates were eligible for the study. Among these neonates 131 were evaluated for early onset sepsis and 173 were evaluated for late onset sepsis. There were 84 neonates in culture positive sepsis group and 60 neonates in probable sepsis group and 160 neonates in no sepsis group, which were considered as controls (Fig. 1).

Among the culture positive cases, gram negative organisms (73%) accounted for majority of the cases followed by gram positive (19%) and fungus (8%). The common gram negative organisms cultured were *non-fermenting gram negative bacilli* (NFGNB) (21%), *klebesiella* (18%), *pseudomonas* (8%) and *E coli* (6%). Among the gram-positive organisms, *coagulase negative staph aureus* (14%), *staph aureus* (9%), *streptococcus hemolyticus* (1%) and *enterococcus* (2%) accounted for majority of cases. The baseline characteristics are shown in Table 1.

**Table 1:** Baseline variables and Sepsis screen.

		Sepsis group		Controls	P value
	Total (n = 304)	Culture positive Sepsis (n = 84)	Probable sepsis (n = 60)	No sepsis (n = 160)	
Age (days) ^#^	4.5 (2–10)	7 (4–13)	7.5 (3–14)	3 (2–5.75)	<0.01
Weight (Kg) ^#^	2.5 (1.96–2.8)	2.420 (1.78–2.7)	2.5 (2–2.86)	2.57 (2.1–2.8)	0.25
Gestation (weeks) ^#^	38 (36–39)	37 (36–38)	37 (36–39)	38 (37–39)	0.06
Male ^*^	155 (51)	44 (52)	23 (38)	88	0.08
Prematurity^*^	79 (26)	29 (34)	17 (28)	33	0.08
Anemia (Hb < 10 g/dl) ^*^	18 (6)	15 (18)	2 (3)	1	<0.01
Hemoglobin (g/dl) ^#^	13.9 (12.2–16)	12.5 (10.8–14.5)	13.9 (12.8–16.1)	14.5 (13.20–16.87)	<0.01
Total leukocyte count (cu/mm^3^)^#^	10500 (7625–15650)	11650 (3762.50–17575)	16200 (11600–18690)	9800 (8400–11300)	0.81
Absolute neutrophil count (cu/mm^3^) ^#^	5800 (3812–10987)	7400 (1870–13252)	11935 (8070–14200)	5090 (4025–6222)	0.20
I/T ratio ^#^	1 (1–2)	2 (2–2)	2 (1–2)	1 (1–1)	<0.01
Leukopenia (cu/mm^3^) ^*^	34 (11)	25 (30)	8 (13)	1	<0.01
Neutropenia^*^	27 (9)	20 (24)	7 (11)	0	<0.01
Thrombocytopenia ^*^	50 (16)	42 (50)	6 (10)	2	<0.01
Peripheral smear s/o sepsis ^*^	87 (28)	65 (77)	18 (30)	4	<0.01
CRP ( > 10 mg/dl)^*^	134 (44)	78 (93)	54 (90)	2 (1)	<0.01
Culture positive sepsis ^*^	59 (19)	59 (70)	0	0	<0.01
CRP^#^	6 (6–53)	75.1 (30.85–100)	39.9 (18.37–70.97)	6.0 (6–6)	<0.01

^#^Median(IQR), ^*^N(%).

Among the neutrophil VCS parameters, comparing culture positive sepsis with no sepsis group, mean values of all the parameters were significantly different between the two groups. When probable sepsis and no sepsis group were compared, only MNV and VDW were significantly different between the groups with higher values of MNV and VDW in probable sepsis group (Tables 2 and 3).

**Table 2:** Diagnostic accuracy of Neutrophil VCS parameters (Culture positive sepsis vs no sepsis).

	AUC	Cut off value (au)	Sensitivity	Specificity	PPV	NPV	LR+	LR−
MNV	0.99	157.9	97%	96%	92%	98%	24.25	0.03
MNC	0.59	137.9	63%	57%	43%	74%	1.46	0.64
MNS	0.28	120.3	47%	32%	26%	53%	0.69	1.65
VDW	0.91	31.0	88%	74%	63%	92%	3.38	0.16
CDW	0.60	12.1	61%	46%	36%	69%	1.12	0.84
SDW	0.61	12.7	70%	53%	43%	77%	1.48	0.56

**Table 3:** Neutrophil VCS parameters.

	TOTAL (n = 304)	Sepsis (n = 84)	Probable sepsis (n = 60)	No sepsis (n = 160)	P value (No sepsis vs probable sepsis)	P value (No sepsis vs culture positive sepsis)
MNV	156.4 (149.6–169.2)	180.3 (169.2–194.2)	163.1 (159.4–169.1)	150.0 (146.3–153.5)	<0.01	<0.01
MNC	138.1 (134.1–146.5)	141.7 (132.7–150.3)	138.3 (134.7–146.7)	137.2 (134.1–144.7)	0.92	0.03
MNS	122.9 (118.9–128.3)	199.6 (117.3–122.8)	122.2 (188.9–128.2)	125.7 (119.8–129.7)	0.53	<0.01
VDW	30.3 (28.5–36.7)	38.5 (35.7–41.9)	34.5 (29.8–36.7)	28.7 (27.3–30.2)	<0.01	<0.01
CDW	12.6 (10.6–14.2)	13.7 (11.0–18.3)	11.3 (10.2–12.8)	12.6 (10.6–13.7)	0.05	0.02
SDW	13.2 (12.1–14.5)	13.6 (12.4–15.7)	13.3 (12.1–13.8)	12.7 (12.1–14.3)	1.00	0.01

**Figure 2:**
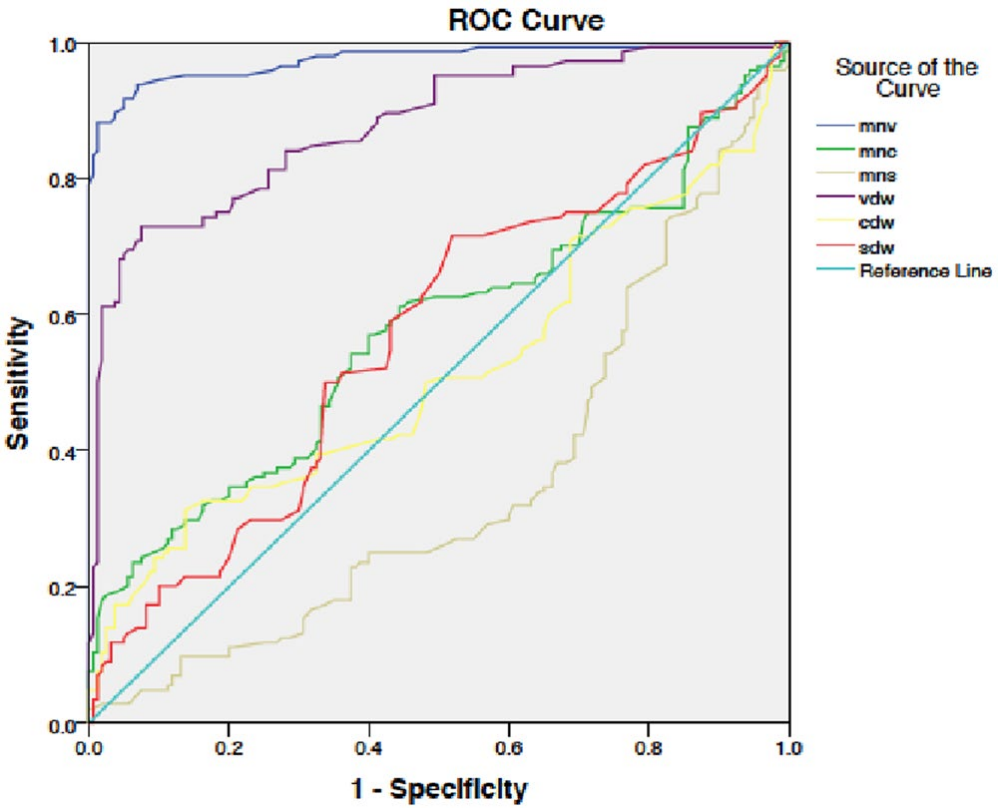
ROC curve showing various neutrophil VCS parameters (Sepsis group vs No sepsis).

**Figure 3:**
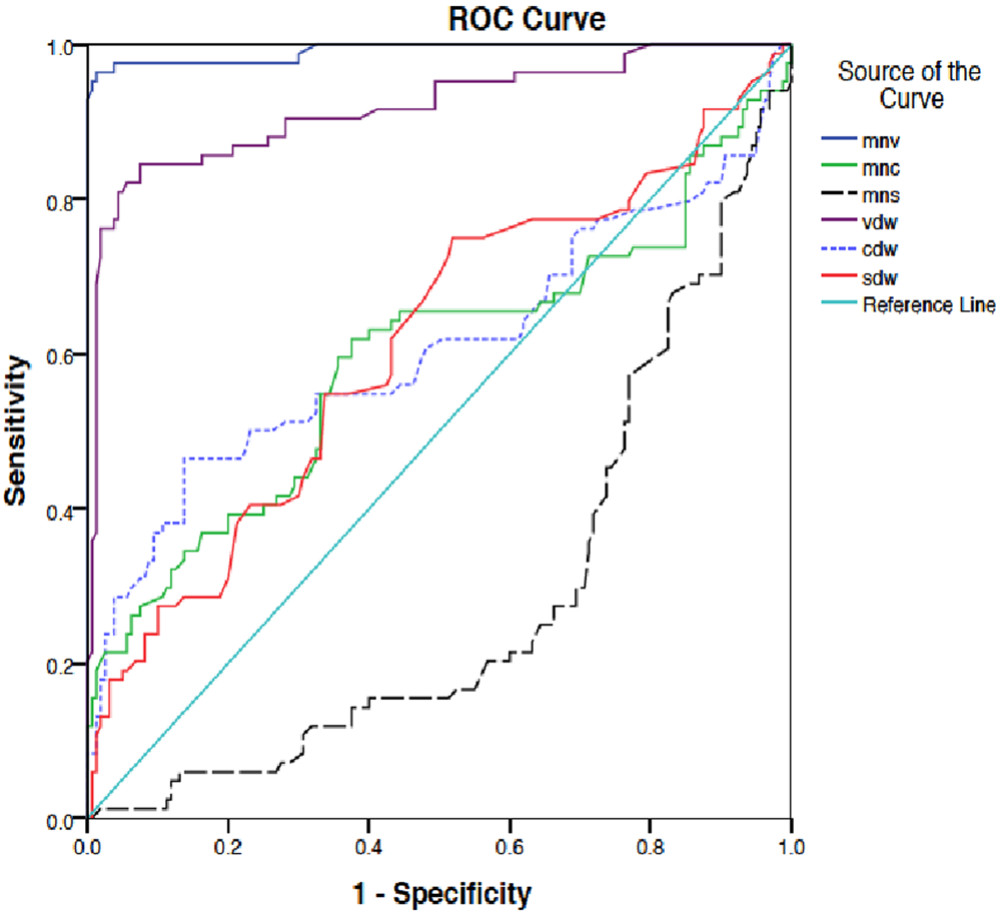
ROC curve showing various neutrophil VCS parameters (Culture positive sepsis vs No sepsis).

Among all the parameters, MNV and VDW had the highest sensitivity and specificity for detecting the sepsis state (97%, 96% and 88%, 74% respectively). The area under curve (AUC) in ROC curve was more than 0.8, being 0.99 and 0.91 with cut off value of 158 and 31 for MNV and VDW respectively. MNV and VDW had overall higher sensitivity and specificity compared to other parameters when overall sepsis group was compared with controls (Tables 2 ,4) (Figs. 2 and 3).

**Table 4:** Diagnostic accuracy of Neutrophil VCS parameters (Sepsis vs controls).

	AUC	Cut off value (au)	Sensitivity	Specificity	PPV	NPV	LR+	LR−
MNV	0.97	156.3	95%	86%	85%	95%	6.78	0.05
MNC	0.57	137.3	62%	55%	55%	62%	1.37	0.69
MNS	0.35	120.2	51%	45%	45%	50%	0.92	1.08
VDW	0.87	30.0	82%	74%	73%	82%	3.15	0.24
CDW	0.51	11.3	60%	48%	50%	57%	1.15	0.83
SDW	0.56	12.5	71%	50%	56%	66%	1.42	0.58

No statistically significant differences were found in Neutrophil VCS parameters between early and late onset sepsis. There was also no difference between the groups when neutrophil VCS parameters were compared based on etiology of sepsis (gram positive vs gram negative vs fungal sepsis). Among the routine sepsis screen tests and other categorical parameters, Age, elevated CRP, haemoglobin, I/T ratio, platelet count were significantly different between culture positive sepsis and no sepsis group.

**Figure 4:**
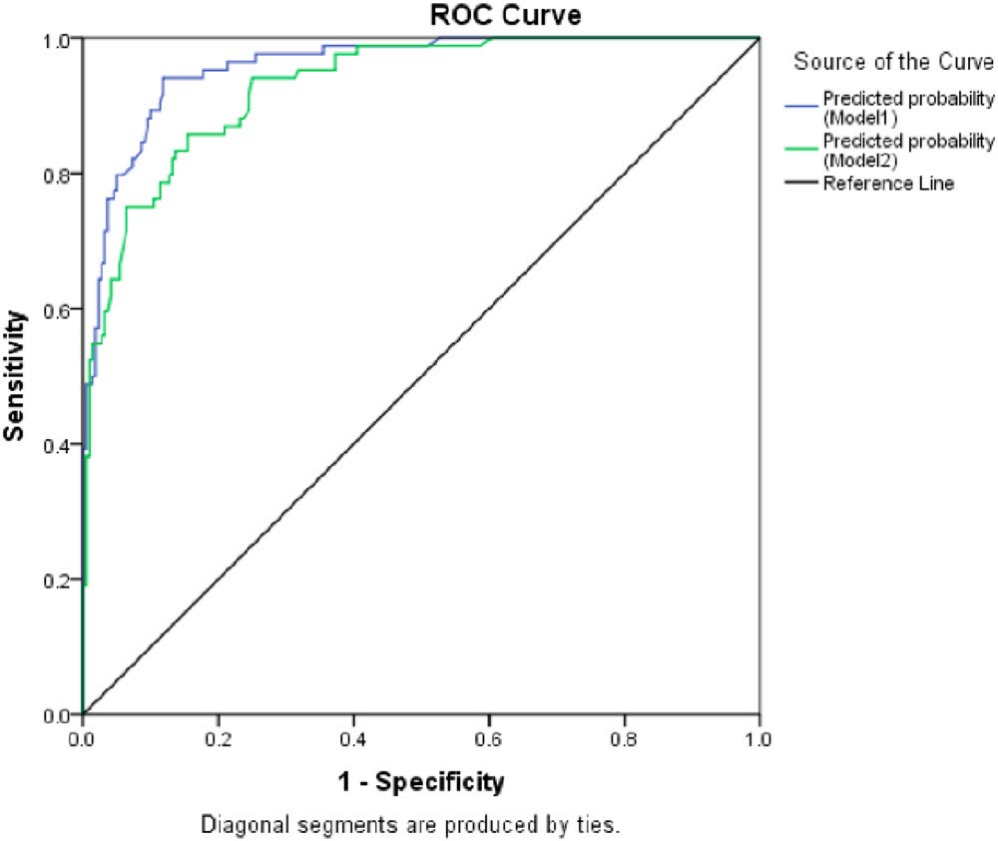
ROC curve showing composite Parameters (culture positive sepsis vs no sepsis).

We also analysed best of the 2 VCS parameters (MNV and VDW) with other haematological parameters to improve the diagnostic accuracy. There was improvement in sensitivity from 88% to 94% when VDW was combined with other haematological parameters (Table 5) (Fig. 4).

**Table 5:** Diagnostic accuracy of composite parameters (Culture positive sepsis vs no sepsis).

	AUC	Sensitivity	Specificity	PPV	NPV	LR+	LR −
MNV + Abnormal leukocyte count + neutropenia+ thrombocytopenia	0.95	95%	83%	67%	97%	5.3	0.05
VDW + Abnormal leukocyte count + neutropenia+ thrombocytopenia	0.92	94%	75%	59%	97%	3.7	0.07

## Discussion

Neonatal sepsis accounts for one of the most common cause of neonatal mortality in india^1^. Sepsis screen, which involves a combination of haematological and laboratory parameters is the most common initial test performed in evaluation of any neonate with suspected sepsis. It has its own drawbacks like poor positive predictive value and less utility in early evaluation^7^. This has led to quest to other markers, which are non specific. Among all the parameters of sepsis screen immature to total neutrophil ratio (I/T ratio) has the best sensitivity^7^.

The volume, conductivity, and scatter (VCS) technology is considered equivalent to microscopic evaluation of peripheral smear and immature to total neutrophil ratio and gives results within minutes^4^. Routine microscopy has many disadvantages like need for expertise, time consuming and inter and intra observer variation^4^.

There are many studies which have evaluated the role of VCS parameters in the diagnosis of sepsis^3–6,8^.

In most of these studies MNV has shown to have maximum diagnostic value. In the study by Abiramalatha *et al*.^6^, a MNV cutoff of >151 showed 71% sensitivity and 71% specificity with an AUC of 0.783 when analysed as a part of initial sepsis screen. Bhargava *et al.*^2^ study showed MNV > 154.2 had sensitivity and specificity of 95% and 82% respectively with an AUC of 0.93. In another study by Celik *et al*., a cut off MNV > 157 had a 79% sensitivity and 82% specificity with an AUC of 0.85^1^. In one of the recent studies by Celik *et al*., MNV was determined as the most useful VCS parameter with highest specificity for neonatal sepsis. The predictive value was lower than that of CRP and procalcitonin in this study^8^.

In our study MNV > 157 had a sensitivity of 97% and specificity of 96% with AUC of 0.99 in cases of proven sepsis and MNV > 156 had 95% sensitivity and 86% specificity with AUC of 0.97 in case of proven and probable sepsis.

Some of the studies have also investigated the use of combination of VCS parameters along with other parameters of sepsis screen, CRP and IL-6^3,6^. This combination increased the sensitivity in these studies for diagnosis of sepsis^3,6,8^. In the present study we also analysed combination of VCS parameters with abnormal blood counts to improve the diagnostic accuracy. Abnormal leukocyte count, neutropenia and thrombocytopenia were combined with MNV and VDW. There was a marginal increase in sensitivity from 88% to 94% when VDW was combined with other haematological parameters without much change in case of MNV.

Routine parameters of sepsis screen such as neutropenia, leukopenia and ITR can also be used as a sepsis screen parameter but these tests have lower diagnostic value when considered alone^7^. Serial sepsis screen and CRPs have a better diagnostic accuracy than a single value but addition of neutrophil VCS parameters increases the diagnostic value to a higher level^6,8^.

In our study MNV has shown to have a very high sensitivity, specificity, PPV (Positive predictive value), NPV (Negative predictive value) and positive LR+ (Likelihood Ratio) in diagnosis of sepsis, especially in case of culture positive sepsis. The utility of this parameter can be beneficial in many cases of sepsis when sepsis screen is equivocal.

In neonates, one of the main reasons for initiation of antibiotics is suspicion of sepsis. Since MNV also has a high negative predictive value a negative test also helps in withholding the unnecessary antibiotic treatment in case of suspected sepsis where risk of sepsis is low.

This is one of the largest study reported on neutrophil VCS parameters in neonatal sepsis. These parameters can be performed with analysers without the need of much expertise and can be analysed quickly. This contrast with sepsis screen where the analysis of parameters like CRP and IT ratio requires some time^9,10^.

## Conclusion

Neutrophil VCS parameters, especially mean neutrophil volume (MNV) which has high sensitivity and specificity can be incorporated with other sepsis screen parameters in diagnosis of neonatal sepsis.
